# Predicting phosphorylation sites using machine learning by integrating the sequence, structure, and functional information of proteins

**DOI:** 10.1186/s12967-021-02851-0

**Published:** 2021-05-24

**Authors:** Salma Jamal, Waseem Ali, Priya Nagpal, Abhinav Grover, Sonam Grover

**Affiliations:** 1 JH-Institute of Molecular Medicine, Jamia Hamdard, New Delhi, India; 2 School of Biotechnology, Jawaharlal Nehru University, New Delhi, India

**Keywords:** Post-translational modification, MRMR, Symmetrical uncertainty, Random forest, Support vector machine

## Abstract

**Background:**

Post-translational modification (PTM) is a biological process that alters proteins and is therefore involved in the regulation of various cellular activities and pathogenesis. Protein phosphorylation is an essential process and one of the most-studied PTMs: it occurs when a phosphate group is added to serine (Ser, S), threonine (Thr, T), or tyrosine (Tyr, Y) residue. Dysregulation of protein phosphorylation can lead to various diseases—most commonly neurological disorders, Alzheimer’s disease, and Parkinson’s disease—thus necessitating the prediction of S/T/Y residues that can be phosphorylated in an uncharacterized amino acid sequence. Despite a surplus of sequencing data, current experimental methods of PTM prediction are time-consuming, costly, and error-prone, so a number of computational methods have been proposed to replace them. However, phosphorylation prediction remains limited, owing to substrate specificity, performance, and the diversity of its features.

**Methods:**

In the present study we propose machine-learning-based predictors that use the physicochemical, sequence, structural, and functional information of proteins to classify S/T/Y phosphorylation sites. Rigorous feature selection, the minimum redundancy/maximum relevance approach, and the symmetrical uncertainty method were employed to extract the most informative features to train the models.

**Results:**

The RF and SVM models generated using diverse feature types in the present study were highly accurate as is evident from good values for different statistical measures. Moreover, independent test sets and benchmark validations indicated that the proposed method clearly outperformed the existing methods, demonstrating its ability to accurately predict protein phosphorylation.

**Conclusions:**

The results obtained in the present work indicate that the proposed computational methodology can be effectively used for predicting putative phosphorylation sites further facilitating discovery of various biological processes mechanisms.

**Supplementary Information:**

The online version contains supplementary material available at 10.1186/s12967-021-02851-0.

## Background

The post-translational modification (PTM) of proteins plays an extremely important role in numerous cellular functions and biological processes [1], including altering proteins’ physiochemical properties, conformation, localization, and enzymatic activity; it also plays an important role in several other processes, such as cell signaling, regulation of gene expression, and cellular metabolism, to name a few [2]. Over 200 diverse PTMs have been recognized [3], of which phosphorylation is the most abundant and well-established PTM in eukaryotes and is crucial to almost all aspects of cell life.

Protein phosphorylation is a rapid process involved in signal transduction pathways, cell proliferation and differentiation, metabolic activities, regulating protein functions, DNA replication, apoptosis, etc. [4–6]. Although PTMs are essential to homeostasis in biological systems, an individual PTM can also disrupt the regulation of complex protein networks, further affecting protein function and leading to many diseases and disorders (most of which are related to aging and dementia) [7]. The most common example of such disruption is the extensive phosphorylation of tau proteins in neurofibrillary tangles, which leads to neurodegenerative disorders such as Alzheimer’s disease and Parkinson’s disease. Over-phosphorylation of tau proteins promotes their aggregation and reduces the stability of microtubules, kinase, and phosphatase activity, thus exacerbating neurotoxicity [8]. The identification and elucidation of the role of PTMs is therefore required to better understand the molecular mechanisms of modified proteins, which could lead to the development of potential disease interventions and treatments.

During phosphorylation, a phosphate group is added to the side chain of an amino acid (AA)—mainly serine (Ser), threonine (Thr), or tyrosine (Tyr), but to a lesser extent to arginine, lysine and histidine residues [9]. This reaction is catalyzed by kinase enzymes and is reversible, during which phosphate groups are removed by specific protein phosphatases [10]. Phosphorylation of an AA residue by protein kinase is also known to depend on the neighboring AAs [11]. Over the years, PTMs have been identified experimentally using biological methods, including mass spectrometry and site-directed mutagenesis [12]. Although these techniques provide a vast amount of data when operated in a high-throughput manner, they are laborious, costly, time-consuming, and often produce false positives and false negatives. A large number of PTMs thus remain unidentified or misclassified, and the associated mechanisms in context of cellular and biological processes are overlooked [13]. The computational prediction of protein phosphorylation sites appears to be a promising alternative strategy for reducing the associated costs and time. The preliminary prediction of phosphorylation sites together with experimentally identified PTMs would expand our knowledge of the molecular mechanisms behind phosphorylation events and aid in protein functional characterization.

Around 40 computational methods have been developed to predict protein phosphorylation sites [14]. For example, Maiti et al. proposed an approach that uses sequence-environment-specific, geometric, and evolutionary information-based features to identify phosphorylation sites using the LightGBM algorithm [13]. Other machine-learning (ML)-based approaches, such as PhosPred-RF [11] and PhosphoSVM [15], use only sequence-based features for predictions based on random forest (RF) and support vector machine (SVM), respectively. NetPhos [16] uses a combination of sequence and structural features for independent and kinase-specific predictions, whereas PPRED uses only evolutionary information to classify phosphorylation sites [17]. PhosphoPredict [10] also uses a combination of sequence and functional features to decipher kinase-specific substrates and their related phosphorylation sites. Other methods that use general and kinase-specific sequences for prediction are MusiteDeep [18], DeepPhos [19], Scansite [20], KinasePhos2.0 [21], and GPS [22].

In the present study, RF and SVM algorithm-based learning were used to predict pS, pT, and pY residues from the protein sequences. A multitude of features, including sequence-based features, physicochemical-property-based (PP) features, structural features (SF), functional features (FF), and functional annotation (FA) represented sequence fragments around phosphorylation sites. A two-step feature selection approach and a minimum redundancy maximum relevance (mRMR) approach, followed by symmetrical uncertainty (SU), produced the most informative features for training the classifiers.

## Methodology

**Fig. 1:**
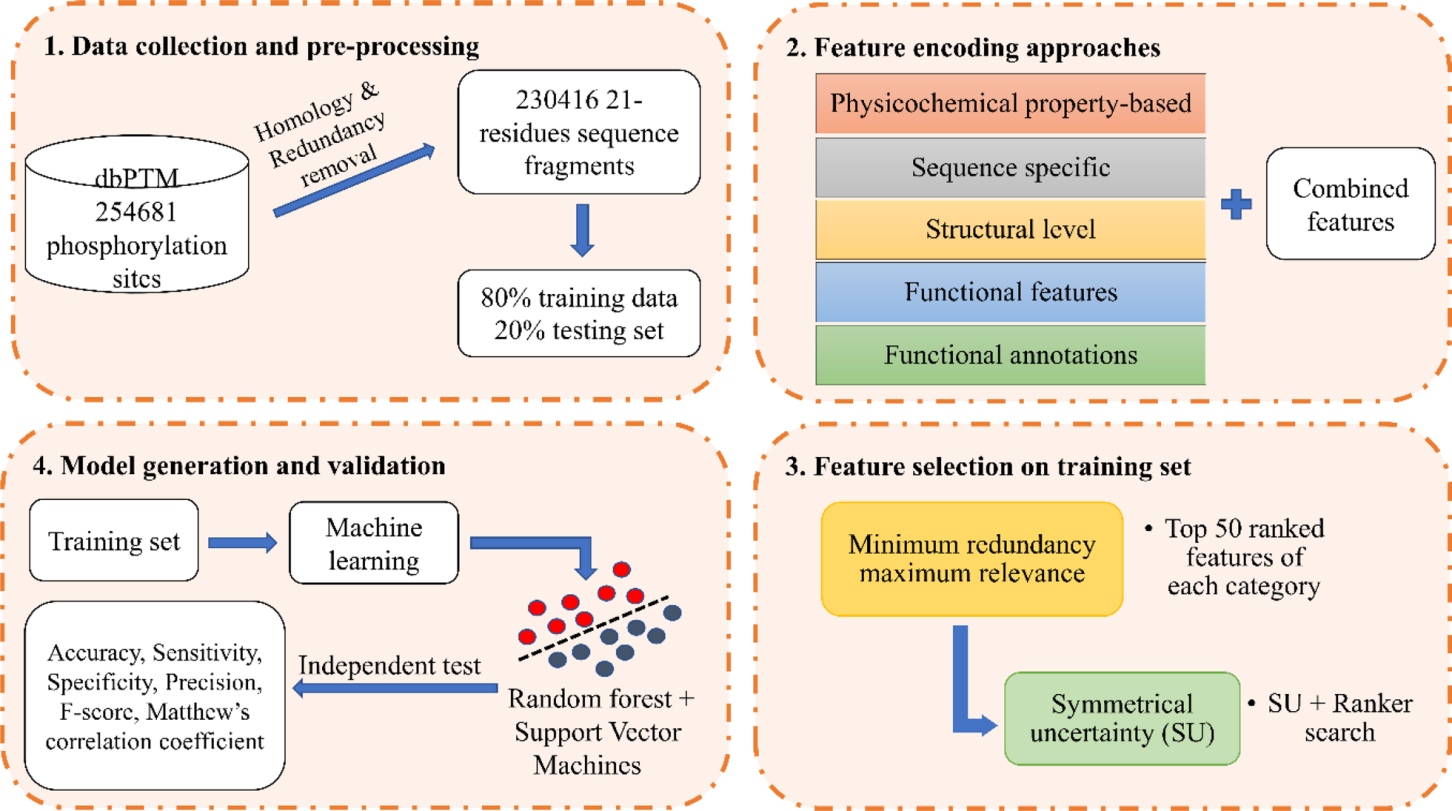
Overall workflow of the proposed approach for prediction of phosphorylation sites

Figure 1 depicts the overall workflow of the proposed approach for prediction of phosphorylation sites.

### Data collection and pre-processing

The data comprising of experimentally validated phosphorylation sites, pS, pT and pY was extracted from publicly available dbPTM [23] database. This database provides information integrated from several databases that include Phospho.ELM [12], HPRD [24], PhosphoNET [25] amongst others. A total of 256,481 experimental phosphorylation sites belonging to human category were obtained from dbPTM following which after the removal of homologous and redundant sequences, 230,416 phosphorylation sites remained encoded in 21-residues sequence fragments. Finally, a total of 384,591, 61,004 and 125,142 protein sequence fragments were obtained for pS, pT and pY, respectively. The peptides having the central S, T or Y residue phosphorylated were considered as positive dataset and the other non-phosphorylated sites were labelled as negative dataset. The final datasets were divided into 80% training data for learning and 20% testing set for validation using an in-house Perl script. Feature selection and ML model generation was done using training dataset followed by five-fold internal cross validation for performance’s evaluation of the trained classifiers. Further assessment of ML models was carried out by validation with an independent testing dataset using various statistical measures.

### Feature encoding approaches

Each peptide sequence was converted into numeric feature vectors which were then used to generate ML models. For each positive and negative sequence fragment, the features were extracted for central residue as well as ten neighboring residues upstream and downstream of central site to capture the local information of the phosphorylation site. A total of 1404 features categorized into groups, PP features, SF, SSF, FF and FA were used to represent each AA in the present study. Additional file 1 provides a comprehensive list of all the extracted features.

### Physicochemical property-based features

In order to capture the environment around the central phosphorylated residue, AAindex database [26] was used to obtain numeric vectors representing physicochemical and biochemical properties of each AA. Seven properties used in the present study covered AA composition (AAC), average flexibility indices, hydrophobicity indices, net charge, partition coefficient, residue volume and molecular weight. Each sequence fragment with a length of 21 residues was represented by 7 properties resulting in a 147 (21 × 7 = 147) dimensional vector.

### Sequence-based features

Previous studies have shown that neighboring AAs of phosphorylated residue are the key sequence-based features for the prediction of phosphorylation sites [27]. Binary-encoding (BE) method was used in which each AA corresponded to a 20-dimensional binary vector comprised of elements, ‘0’ and ‘1’. For example, Alanine is represented as a vector ‘10000000000000000000’, Serine as ‘00000000000000010000’ and so on and thus we obtained a 420-dimensional vector (21 × 20 = 420).

### Structural level features

Three SSF used in the present study are accessible surface area (ASA), secondary structure (coil, helix and strand) and disordered regions. ASA gives an estimate of accessibility of an AA to solvent in a protein thus giving crucial information on the protein structure [28]. Thus, ASA of individual AA residues for each protein sequence was obtained from AAindex. Another factor that gives insights about protein structure is the secondary structural configuration of AAs. A neural network-based prediction tool, PSSpred [29] was used for secondary structure prediction of all the AAs in each protein sequence fragment. It has been observed that phosphorylation sites are usually located in disordered regions, which makes protein disorder an important feature for predicting phosphorylation sites. The native disorder information was predicted using IUPRED2A which is an energy estimation method taking into account differences between ordered and disordered regions [30]. The scores between, 0 and 1 were obtained amongst which residues above 0.5 score were considered as disordered and the others below 0.5 were labelled to be in ordered region. In all, 5 feature vectors denoted structural features resulting in a 105 (21 × 5 = 105) dimensional vector representing each sequence fragment.

### Functional features

The functional information incorporated for prediction of phosphorylation sites include gene ontology terms (1) biological process (BP), (2) molecular function (MF) and (3) cellular component (CC); (4) protein functional domain data from InterPro [31]; and (5) KEGG pathway [32] information through DAVID tool [33] which is a gene functional classification tool. A total of 846 FF including 555 GO terms, 177 functional domain types and 114 terms denoting KEGG pathways were acquired and each AA was encoded into ‘0’ and ‘1’ according to the absence and presence of FFs, respectively.

### Functional annotations

Using functional annotation tool available from DAVID, functional properties belonging to two categories, UP_SEQ_FEATURE and UP_KEYWORDS, were retrieved. A total of 526 types of protein functional annotations were obtained where an AA residue was denoted by ‘1’ if it had annotation for a particular function and ‘0’ if it was not linked with a specific function.

### Combined features models

In order to enhance the prediction performance, all the groups, PP-based, SF, SSF, FF and FA features were pooled resulting in a total of 1404 features to generate learning models.

### Feature selection

Feature selection methods were used to choose the most significant and informative features while minimizing the redundancy in the data thereby reducing its dimensionality and computational time and further improve model performances [34]. In the present study, feature selection was performed at two levels: mRMR approach followed by SU attribute selection method.

### Minimum redundancy maximum relevance

mRMR is a widely used feature selection method based on mutual information. This approach ranks the features taking into consideration their importance to the classification variable along with the redundancy amongst the features themselves. A higher ranked attribute indicates its high correlation with the classification variable and least redundancy [35]. Top 50 ranked features of each category were selected as the most contributing features.

### Symmetrical uncertainty

SU attribute evaluation method weighs the merit of an attribute by determining its uncertainty with reference to other sets of attributes [36]. SU can be calculated by the following equation:

$$SU \left(X,Y\right)= \frac{2*IG (X|Y)}{H \left(X\right)+H (Y)},$$

where IG stands for information gain, H denotes entropy and X and Y represent attributes [37].

Considering the ability of this method to balance the biasness of information gain towards certain attributes [38], SU is a method of choice for a plethora of feature selection tasks [39, 40]. Weka software [41] was used to implement SU in combination with Ranker search which returned a list of top ranked attributes followed by the less significant ones and lastly the least important.

### Training machine learning models

#### Random forest

RF is broadly used ML algorithm used for solving classification problems and making predictions [42–44]. This algorithm is based on the ensemble of decision-making trees which yield individual outputs and the most common output of the model is considered as final RF prediction. The node of the tree and the subset of features used for generating trees is chosen randomly [45]. RF has many advantages which made it suitable for use in the present study. It is considered as a highly accurate learning classifier which can efficiently handle large dimensional datasets, deals with overfitting and does not consume a lot of time for training and prediction amongst many others [42, 46]. In this study, the RF models were trained using RandomForest package available from Weka [41].

#### Support vector machine

SVM is one of the most extensively applied ML algorithm in various computational studies involving classification and regression tasks [44, 47–50]. This algorithm finds an optimal hyperplane in a high-dimensional feature space using a kernel and then categorizes the input vectors into two classes [51]. The aim is to maximize the gap between input vectors of both the classes. In the present study, SVM was used with radial basis function (RBF) kernel implemented using Weka [41].

### Model performance evaluation

To assess the prediction performance of the ML models generated in this work, several statistical measures, accuracy (ACC) that is proportion of correct positive and negative predictions, sensitivity (SN) or true positive rate (TPR), specificity (SP) which is percentage of correctly predicted non-phosphorylated sites, precision (PRE), F-score and the Matthew’s correlation coefficient (MCC) were used [10]. These are defined in the following lines:

$$ACC= \frac{(TP+TN)}{(TP+TN+FP+FN)},$$

$$SN= \frac{TP}{TP+FN},$$

$$SP= \frac{TN}{TN+FP},$$

$$PRE= \frac{TP}{TP+FP},$$

$$F{\text -}score=2 \times \frac{TP}{2TP+FP+FN},$$

$$MCC= \frac{(TP\,\,X\,\,TN)-(FP\,\,X\,\,FN)}{\sqrt{\left(TP+FN\right)X\,\, \left(TP+FP\right)X\,\, \left(TN+FN\right)X\,\,(TN+FP)}},$$

where TP, TN, FP and FN correspond to the numbers of true positives, true negatives, false positives and false negatives. Furthermore, the area under the curve (AUC) calculated from receiver operating characteristic (ROC), which is a plot of SN vs 1 minus SP, was used for evaluating model performances [10].

## Results

### Input data transformation

In the case of each protein sequence, centered residue was flanked by 10 AAs in forward and backward directions (± 10); the problem we addressed was whether the central residue acts as a phosphorylation site and belongs to class 1 or 0 (a non-phosphorylation site). In the present study, 61.2% instances were phosphate-binding and 38.7% belonged to class 1 (and were thus non-phosphate-binding). Totals of 134,584, 57,440, and 36,347 pS, pT, and pY, respectively, belonged to the positive (class 1) dataset, while pS 108,975, pT 27,415, and pY 316 sequence fragments belonged to the negative dataset (class 0). Table 1 provides the number of positive and negative phosphorylation sites in training and testing datasets.

**Table 1:** The number of phosphorylation sites

	Training set		Testing set	
	Positive	Negative	Positive	Negative
Serine	107,668	87,180	26,916	21,795
Threonine	45,952	21,932	11,488	5483
Tyrosine	29,078	253	63	7269

### Evaluating contribution of different feature encoding schemes

With regard to post-mRMR and SU attribute selection, of the PP-based features, AAC, molecular weight, residue volume, flexibility, and partition coefficient of predominantly AA11 turned out to be highly significant for classification, followed by the hydrophobicity of the AAs around the central residue. These features have already been shown to be relevant for discriminating between phosphorylated and non-phosphorylated sites [13, 18]. Table 2 lists the initial number of different features types used to encode sequence fragments.

**Table 2:** Initial number and types of different features used to encode sequence fragments

Feature types	Features	Number
Physicochemical property-based	Amino acid composition, average flexibility indices, hydrophobicity indices, net charge, partition coefficient, residue volume and molecular weight	147 (21 × 7)
Sequence-based	Binary-encoding	420 (21 × 20)
Structural level	Accessible surface area; secondary structure (coil, helix and strand) and disordered regions	105 (21 × 5)
Functional features	Gene ontology (GO) terms (1) biological process (BP), (2) molecular function (MF) and (3) cellular component (CC); protein domain and KEGG pathway	555 GO, 177 domain, 114 KEGG pathway
Functional annotation	UP_SEQ_FEATURE and UP_KEYWORDS	526

In SS features, the secondary structural conformation of AAs demonstrated maximum involvement in S, T, and Y phosphorylated sites prediction, followed by the disordered region, in accordance with several previous studies [10, 27]. Of all the SS features used to train the ML models, ASA contributed least.

The highly informative features representing functional information, included only GO and KEGG pathway terms, whereas the data for the domains related to phosphorylated sequence fragments did not contribute at all to the prediction of PTM sites. In GO terms, the BP class majorly influenced the prediction of phosphorylation sites followed by CC terms. The commonly influencing ‘KEGG pathway terms’ were B cell receptor signaling pathway, endometrial cancer, prostate cancer, small-cell lung cancer, non-small-cell lung cancer, melanoma, renal cell carcinoma and platelet activation, which has been shown to play a role in cancer and neurodegenerative disorders [52, 53].

In the case of FA, the terms crucial for pS and pT prediction were “compositionally biased region: Ser/Thr-rich,” “endoplasmic reticulum,” “short sequence motif: nuclear export signal,” “domain: TSPtype-13,” and “metal ion-binding site: Divalent metal cation1” and “cation 2”. In order to determine the most significant group, PP, sequence-based features, SSF, FF and FA feature groups were combined and compared. Additional file 2 presents all of the features obtained using the two-step, mRMR, and SU feature selection approach for predicting pS/pT/pY sites.

### Performance evaluation of ML models using independent testing data

**Fig. 2:**
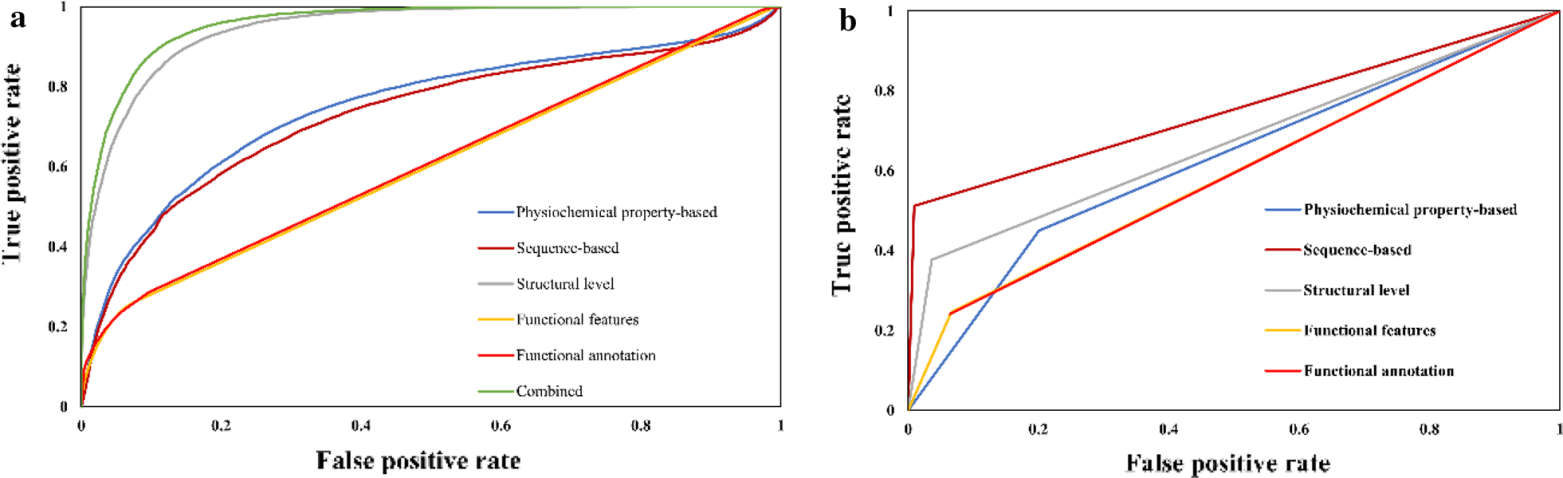
ROC curve on (**a**) random forest (**b**) Support vector machine models using independent test set for Serine phosphorylation site prediction

**Fig. 3:**
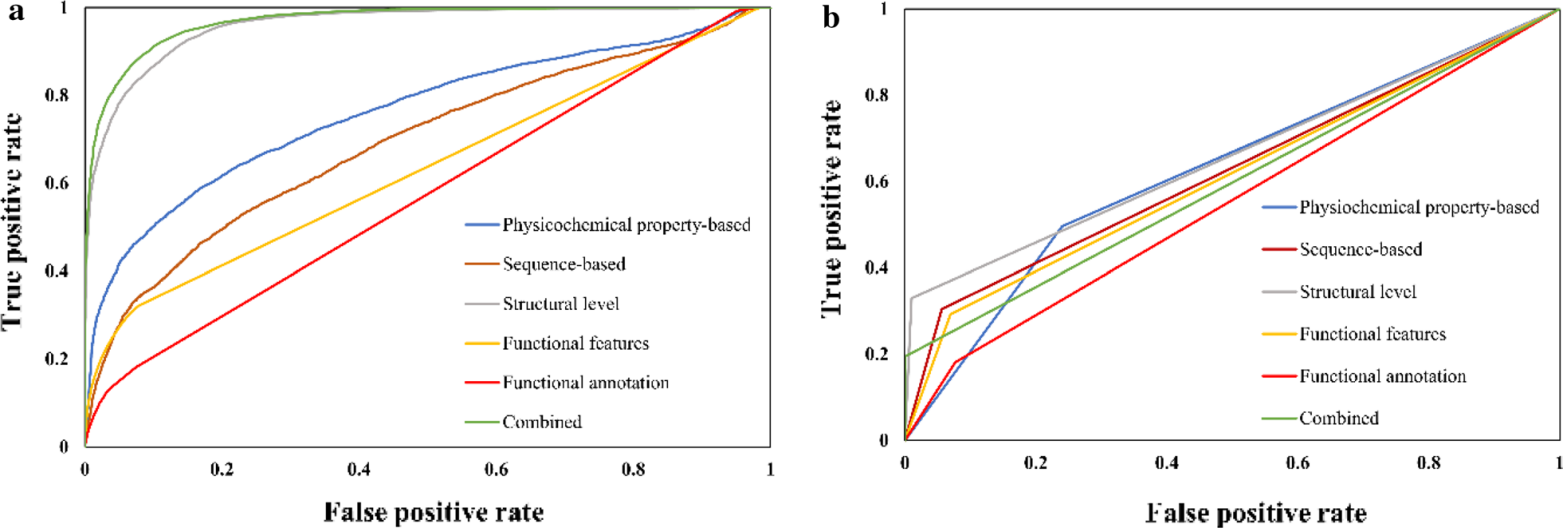
ROC curve on (**a**) random forest (**b**) Support vector machine models using independent test set for Threonine phosphorylation site prediction

**Fig. 4:**
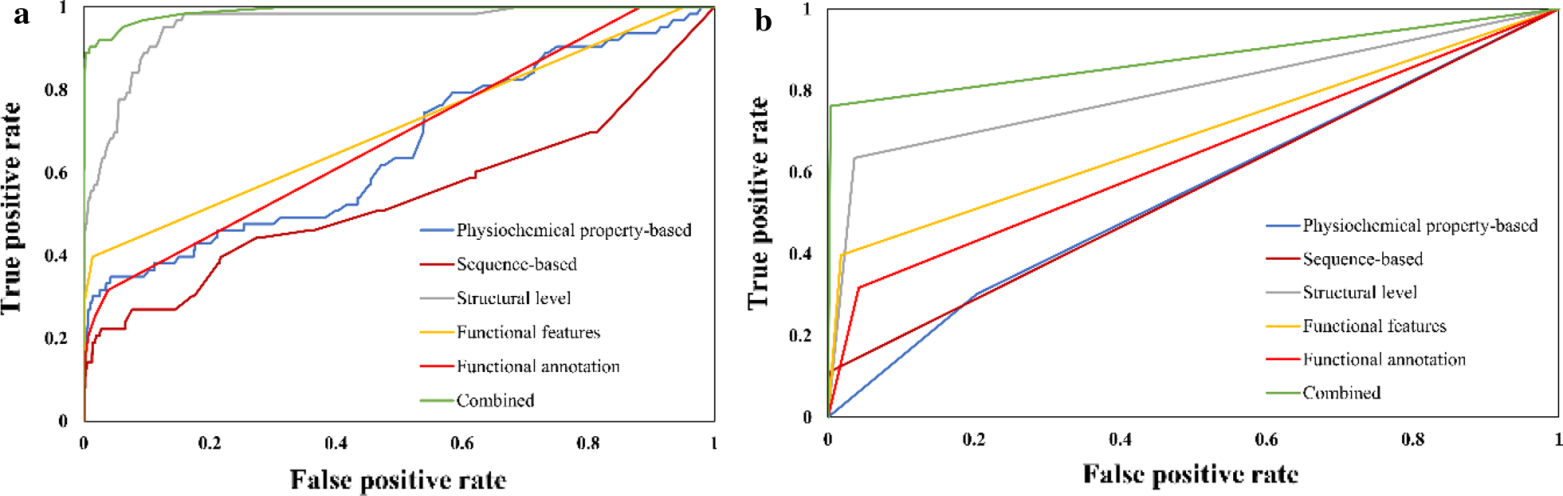
ROC curve on (**a**) random forest (**b**) support vector machine models using independent test set for Tyrosine phosphorylation site prediction

Using individual and combined-feature encoding schemes, two extensively applied algorithms, RF and SVM, were used to generate learned models. The highest AUC values were obtained for the RF model, based on combined feature groups for pS (Fig. 2), pT (Fig. 3), and pY (Fig. 4) (0.95, 0.97, and 0.99, respectively). SVM models also had comparative AUC values, 0.89 for S, 0.59 for T, and 0.87 for Y phosphorylated sites. Furthermore, the RF and SVM ML models generated using secondary structural information produced the second-highest AUC values, in accordance with our feature selection results, which indicated the maximum contribution of SSF to the prediction of pS/pT/pY sites. In addition to the highest AUC values, the combination and the SSF ML models also produced good values for other statistical measures, including ACC, SN, SP, PRE, F-score, and MCC (Tables 3, 4, 5). Moreover, the confusion matrix for all the RF and SVM models generated in the present study have been provided in Additional file 3. All the RF and SVM models generated for pS, pT, and pY prediction have been provided as Additional files 4–39.

**Table 3:** Performance comparison with individual and combined feature encoding schemes for pS site prediction on the independent dataset

Attributes	Methods	Accuracy (%)	Sensitivity (%)	Specificity (%)	Precision (%)	F-measure (%)	MCC	AUC
Physiochemical property	RF	71.5	79	62.3	72.1	75.4	0.42	0.74
	SVM	64.31	79.9	45.0	64.2	71.2	0.26	0.62
Structure	RF	87.34	86.5	80.6	90.2	88.3	0.74	0.94
	SVM	70.12	96.4	37.7	65.6	78.1	0.43	0.67
Sequence	RF	70.3	80.1	58.2	70.3	74.9	0.39	0.73
	SVM	77.65	99.1	51.2	77.5	83.1	0.59	0.75
Functional features	RF	62.87	93.9	24.6	60.6	73.6	0.26	0.59
	SVM	62.58	93.4	24.6	60.5	73.4	0.25	0.59
Functional annotation	RF	62.75	90.7	28.2	60.9	72.9	0.24	0.60
	SVM	62.50	93.6	24.1	60.4	73.4	0.25	0.58
Combined	**RF**	**89.16**	**89.4**	**88.9**	**90.8**	**90.1**	**0.78**	**0.95**
	SVM	88.50	79.9	99.1	99.1	88.5	0.79	0.89

Performance metrics for best results are highlighted in bold

**Table 4:** Performance comparison with individual and combined feature encoding schemes for pT site prediction on the independent dataset

Attributes	Methods	Accuracy (%)	Sensitivity (%)	Specificity (%)	Precision (%)	F-measure (%)	MCC	AUC
Physiochemical property	RF	77.54	92.1	47.1	78.5	84.7	0.45	0.76
	SVM	67.05	77.7	55.9	64.9	70.7	0.34	0.66
Structure	RF	89.58	94.8	78.8	90.3	92.5	0.75	0.96
	SVM	77.66	99	33	75.6	85.7	0.47	0.66
Sequence	RF	71.79	86	42.1	75.7	80.5	0.31	0.69
	SVM	73.74	94.4	69.5	70.3	79.9	0.16	0.57
Functional features	RF	72.75	92.1	32.3	74.0	82.1	0.31	0.63
	SVM	72.43	93.0	29.3	73.4	82.0	0.29	0.61
Functional annotation	RF	68.5	92.6	18.1	70.3	79.9	0.16	0.57
	SVM	68.34	92.3	31.7	70.3	79.8	0.15	0.55
Combined	**RF**	**90.28**	**97.8**	**74.4**	**88.9**	**93.2**	**0.77**	**0.97**
	SVM	73.96	100	19.4	72.2	83.9	0.37	0.59

Performance metrics for best results are highlighted in bold

**Table 5:** Performance comparison with individual and combined feature encoding schemes for pY site prediction on the independent dataset

Attributes	Methods	Accuracy (%)	Sensitivity (%)	Specificity (%)	Precision (%)	F-measure (%)	MCC	AUC
Physiochemical property	RF	77.19	77.5	46	99.4	87.1	0.05	0.65
	SVM	79.21	79.6	30.2	99.2	88.4	0.02	0.54
Structure	RF	99.3	100	79.4	99.3	99.7	0.43	0.95
	SVM	96.08	96.4	63.5	99.7	98	0.27	0.79
Sequence	RF	69.74	70	41.3	99	82	0.02	0.59
	SVM	99	99.8	11.1	99.2	99.5	0.18	0.55
Functional features	RF	98.09	98.6	39.7	99.5	99.0	0.27	0.70
	SVM	97.73	98.2	39.7	99.5	98.9	0.24	0.69
Functional annotation	RF	95.51	96.1	31.7	99.4	97.7	0.12	0.68
	SVM	95.24	95.8	31.7	99.4	97.6	0.12	0.63
Combined	**RF**	**99.42**	**100**	**63.5**	**99.5**	**99.7**	**0.57**	**0.99**
	SVM	99.46	99.7	23.8	99.7	99.7	0.71	0.87

Performance metrics for best results are highlighted in bold

### Comparison with existing methods

To evaluate prediction performance, four existing kinase-independent tools, PhosPred-RF, PhosphoSVM, PPRED, and iPhos-PseEn, were compared to the proposed method of predicting pS/pT/pY sites. The proposed RF-based method clearly outperformed other existing methods with regard to SN, SP, MCC, and AUC values, which corresponded to 0.89, 0.88, 0.78, and 0.95 for predicting pS; SN, SP, MCC, and AUC corresponded to 0.97, 0.74, 0.77, and 0.97, respectively, for predicting pT; and SN, SP, MCC, and AUC corresponded to 0.10, 0.63, 0.57, and 0.99, respectively, for predicting pY (Table 6).

**Table 6:** Performance comparison of different existing tools for pS/pT/pY site prediction

Phosphorylation site	Methods	Sensitivity (%)	Specificity (%)	MCC	AUC
Serine	PhosPred-RF	79.70	75.00	0.54	0.85
	PhosphoSVM	44.43	94.04	0.29	0.84
	PPRED	32.27	91.6	0.16	0.75
	iPhos-PseEn	79.64	79.78	0.39	–
	**Our RF model**	**89.4**	**88.9**	**0.78**	**0.95**
Threonine	PhosPred-RF	73.80	72.60	0.46	0.81
	PhosphoSVM	37.31	94.99	0.25	0.81
	PPRED	34.32	83.65	0.09	0.65
	iPhos-PseEn	71.51	80.68	0.34	–
	**Our RF model**	**97.8**	**74.4**	**0.77**	**0.97**
Tyrosine	PhosPred-RF	72.70	64.00	0.36	0.76
	PhosphoSVM	41.92	87.34	0.20	0.73
	PPRED	43.04	82.65	0.16	0.70
	iPhos-PseEn	76.18	76.29	0.32	–
	**Our RF model**	**100**	**63.5**	**0.57**	**0.99**

Performance metrics for best results are highlighted in bold

### Evaluation of the proposed models’ performance on experimental phosphorylation sites

Owing to the large number of computational methods proposed to identify probable PTM sites, the dbPTM database offers an experimental dataset as a standard to explore the PTM prediction ability of proposed tools. In the present study, the best-performing RF model was applied to a total of 5787 experimentally validated protein phosphorylation sites acquired from the dbPTM repository. Of these 5787 phosphorylation sites, 4312 sequence fragments had Ser as central phosphorylated residue, 1442 had Thr phosphorylated residue, and 33 fragments had phosphorylated Tyr residue positioned in the center of the AA sequence. The results were in accordance with the performance measures obtained from an independent test set validation with 2824 sites predicted to be phosphorylated by Ser, 1410 by Thr, and all 33 sites to be phosphorylated by Tyr, as the pY RF model had the highest ACC and AUC, followed by pT and pS.

## Discussion

Feature selection results in this study showed secondary structural information to be the top-ranked feature in the case of the pS and pT sites. For pY prediction, most of the PP-based features, AAC, flexibility, hydrophobicity, molecular weight, partition coefficient and residue volume, of the 11th AA were revealed to be of utmost importance, followed by KEGG-pathway-associated terms. The other features responsible for pS and pT site predictions involved GO, KEGG pathway, and FA terms. Amongst the GO terms, for pS and pT prediction, the favored CC terms were integrin complex and ciliary base; BP terms included protein import into nucleus, androgen receptor signaling pathway, intracellular receptor signaling pathway, response to cytokine, cell redox homeostasis, and cellular response to ionizing radiation. For pS prediction, MF terms included fatty-acyl-CoA binding, receptor-signaling protein activity, Rho GTPase binding, SH2 domain binding, and Rac GTPase binding. Histone deacetylase activity was the only MF term contributing to pT prediction. The common KEGG pathway terms contributing towards prediction of pS/pT/pY sites were platelet activation, non-small cell lung cancer, melanoma and prostate cancer. Most of the KEGG pathway terms denoted different types of cancer which makes sense as altered phosphorylation has been strongly linked with cancer [54]. Protein domain information of the FF group appeared to be the least informative and contributed minimum to the prediction of S/T/Y phosphorylated sites. Amongst the FA features, the favorably associated terms for pS/pT/pY sites prediction included “alternative promoter usage,” “DNA recombination,” “repeat: TPR8,” and “transit peptide: mitochondrion.” These events have been associated with phosphorylation which is responsible for various diseases and disorders [55–57].

Further during ML model generation, both of the ML algorithms performed well overall in predicting pS/pT/pY sites; however, RF clearly outperformed SVM in most of the feature group models. The best performance for all three of phosphorylated sites for both RF and SVM was achieved using combined feature groups, thereby demonstrating the necessity and significance of exploiting a variety of feature types for prediction. The results of the evaluation of the model performances on the experimental phosphorylation sites confirm that the proposed method can be employed to distinguish unidentified putative phosphorylation and non-phosphorylation sites. On the whole, these results indicate the importance of using different types of feature encoding schemes and feature selection to acquire a diverse set of extremely informative and relevant features for generating high-performance ML models to predict phosphorylation sites.

## Conclusion

Protein phosphorylation is essential to the regulation of biological processes and disease pathogenesis. Experimental identification of phosphorylation sites is time-consuming and costly, so in this paper we proposed an ML-based computation method for cheaper, swift, and efficient S/T/Y phosphorylation prediction. The proposed RF- and SVM-algorithms-based method considers diverse features, physiochemical properties, sequence environment, secondary structure, functional features (pathway, GO, and protein domain), and functional annotation of protein sequence fragments to predict phosphorylation sites. Through two-level mRMR and SU feature ranking we observed that secondary structural information followed by pathway, GO, and FA terms were the most informative features, whereas protein domain features were the least useful. The proposed method also demonstrated significant improvement in performance metrics in terms of SN, SP, MCC, and AUC prediction compared to other existing kinase-independent computational tools. Furthermore, the proposed method exhibited outstanding performance on experimental phosphorylation sites, thereby indicating that it is a promising method for identifying potential pS, pT, and pY sites and would thus facilitate the prediction of functional PTMs and further biological analyses.

## Supplementary Information

**Additional file 1**: Provides a comprehensive list of all the extracted features.

**Additional file 2**: The different types of features obtained after two-step, mRMR and SU, feature selection approach for pS/pT/pY sites prediction.

**Additional file 3**: Confusion matrix for all the RF and SVM models generated in present study for prediction of Ser, Thr and Tyr phosphorylation sites.

**Additional file 4–39**: All the RF and SVM models generated in present study for prediction of Ser, Thr and Tyr phosphorylation sites.

## Data Availability

The datasets used and/or analyzed during the current study are available from the corresponding author on reasonable request.
