# Supplementary material for: Predicting phosphorylation sites using machine learning by integrating the sequence, structure, and functional information of proteins
Source: J Transl Med. 2021 May 24;19:218. doi: 10.1186/s12967-021-02851-0 (PMC8142496; doi:10.1186/s12967-021-02851-0)
Supplement: Supplementary file 3 — Additional file 3: Confusion matrix for all the RF and SVM models generated in present study for prediction of Ser, Thr and Tyr phosphorylation sites. [file 12967_2021_2851_MOESM3_ESM.docx]

**Additional figure 1**


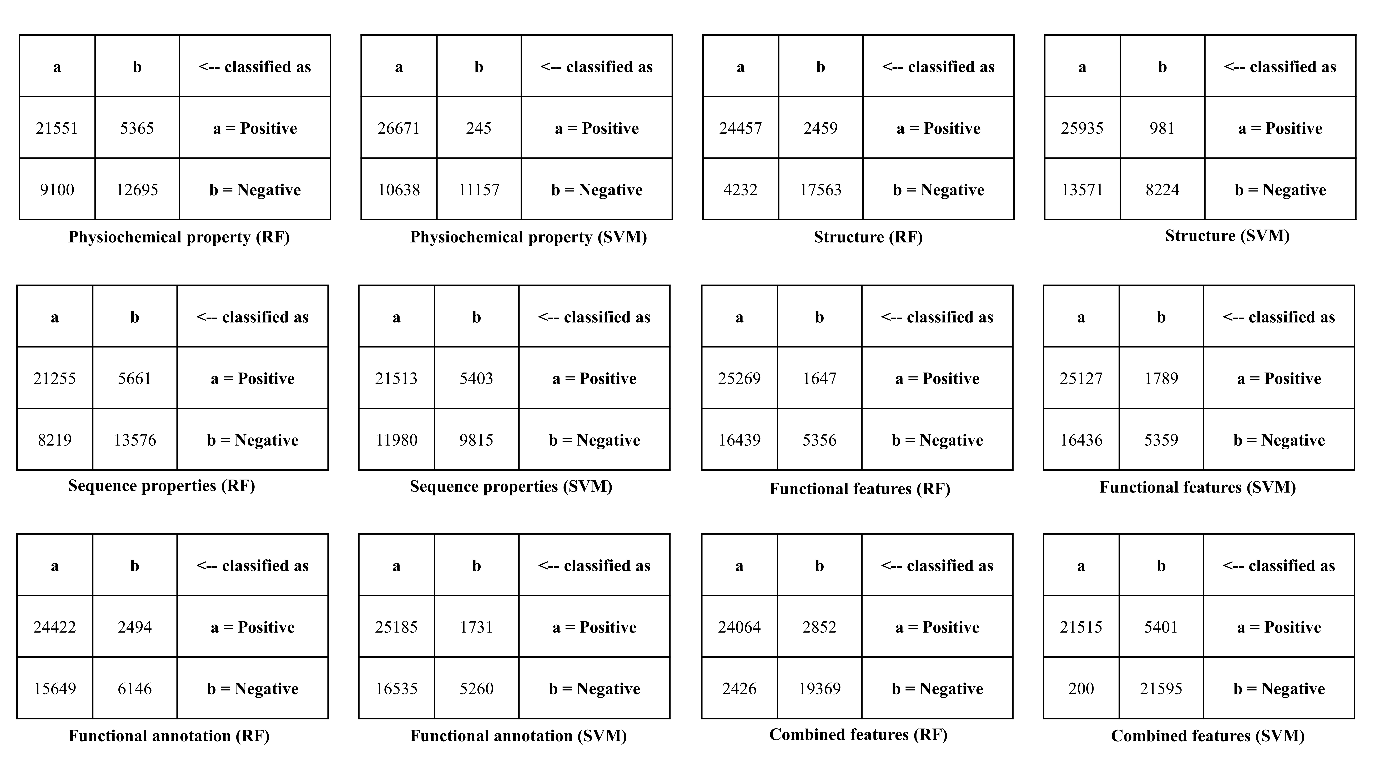


**Additional figure 1.** Confusion matrix for all the RF and SVM models generated in present study for prediction of Ser phosphorylation sites. Top left are True positives. Bottom left are False positives. Top right are False negatives. Bottom right are True negatives.

**Additional figure 2**


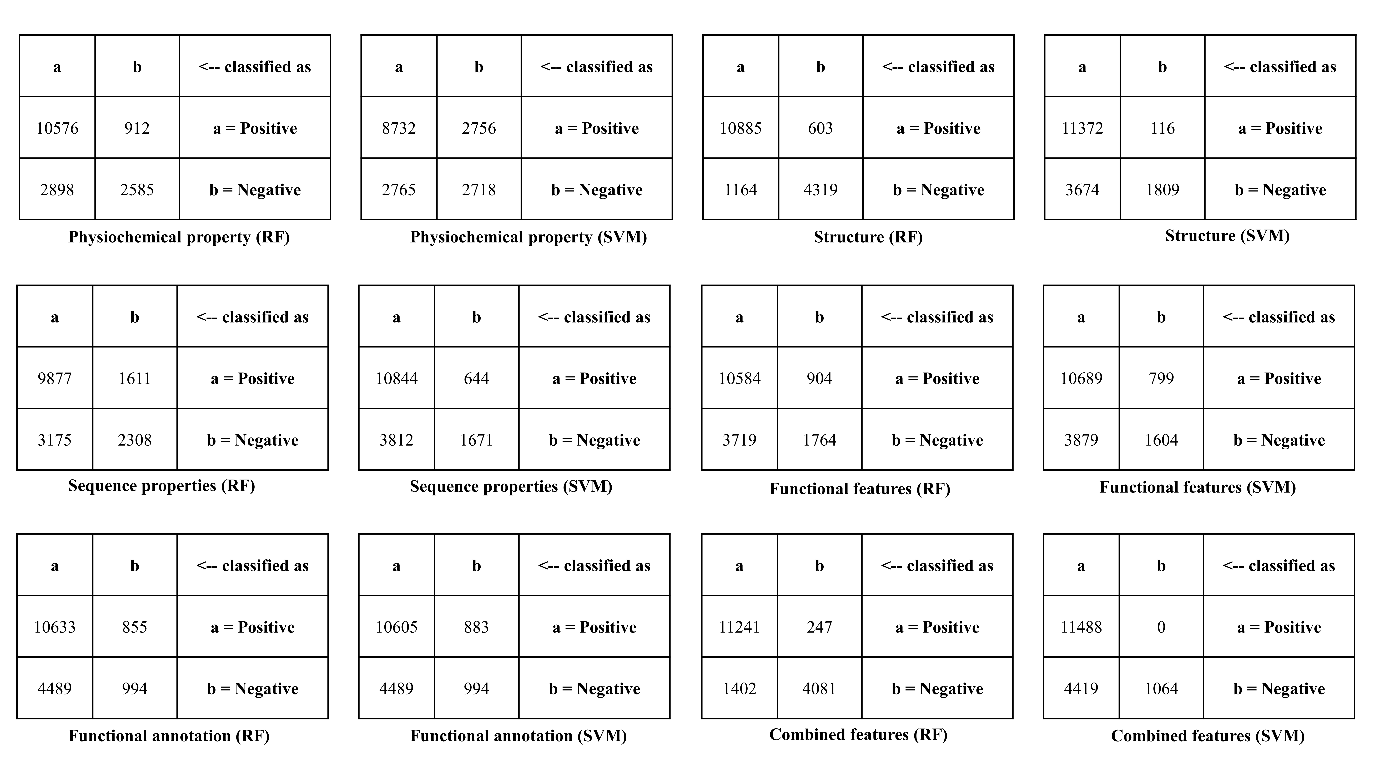


**Additional figure 2.** Confusion matrix for all the RF and SVM models generated in present study for prediction of Thr phosphorylation sites. Top left are True positives. Bottom left are False positives. Top right are False negatives. Bottom right are True negatives.

**Additional figure 3**

**
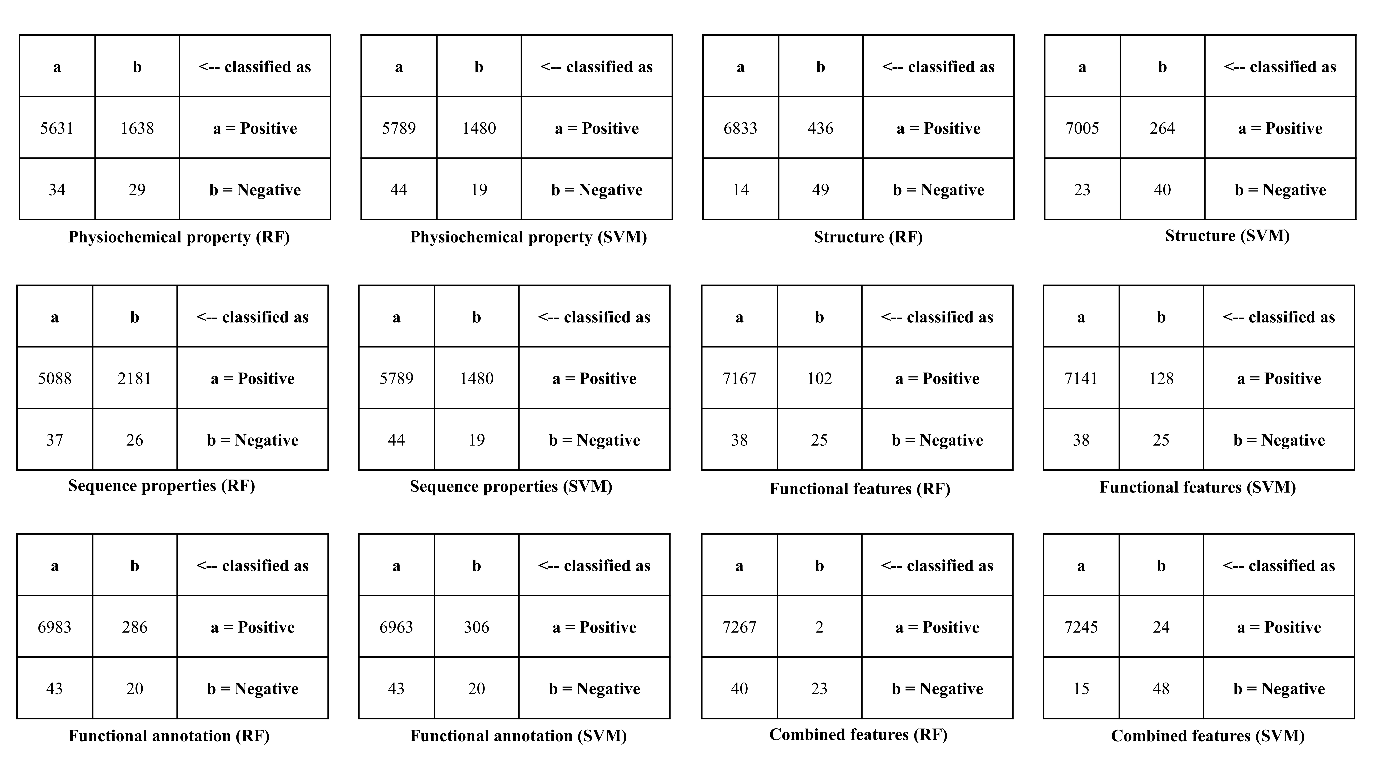
**

**Additional figure 3.** Confusion matrix for all the RF and SVM models generated in present study for prediction of Tyr phosphorylation sites. Top left are True positives. Bottom left are False positives. Top right are False negatives. Bottom right are True negatives.
